# Supplementary material for: Are carbon emissions trading and green financial instruments synergistic? -Comprehensive quantitative research based on content analysis
Source: PLoS One. 2024 Mar 7;19(3):e0298601. doi: 10.1371/journal.pone.0298601 (PMC10919601; doi:10.1371/journal.pone.0298601)
Supplement: S1 Data — (ZIP) [file pone.0298601.s001.zip › Data/地级市Z_F工作报告.zip!/║■▒▒/╧╠─■╩╨/└┤╫╘╣1⁄2╓┌║┼í╛┬φ┐╦╩2╛▌═°í┐_2017.docx]

各位代表：

现在，我代表市人民政府向大会报告政府工作，请予审议，并请各位市政协委员和列席人员提出意见。

一、过去五年工作回顾

过去五年，在省委、省政府和市委的坚强领导下，市政府团结带领全市人民，牢固树立新发展理念，积极适应经济发展新常态，紧紧围绕“建设鄂南强市、打造香城泉都”总目标，攻坚克难、砥砺奋进，圆满完成了预定的目标任务，书写了无愧于时代的光辉篇章。

（一）经济实力大跨越。面对经济下行压力和特大洪涝灾害，我们稳神竞进、激情奋进，奋力建设鄂南强市。地区生产总值2015年跨入千亿俱乐部，2016年预计达到1100亿元，是2011年的1.7倍，年均增长9.7%。固定资产投资、一般公共预算收入、地方税收、规模以上工业总产值实现“四个翻一番”，预计分别达到1418亿元、83亿元、50亿元、1910亿元，年均分别增长20.9%、18.6%、16.9%、15.6%。产值过亿元企业达400家，比2011年增加260家；纳税过千万元企业达32家，比2011年增加15家；市场主体达到15.8万户，比2011年净增5.3万户。

（二）城乡面貌大变化。以新发展理念为引领，打造品牌，提升品质，奋力建设香城泉都。交通条件大幅改善。完成交通投资239.5亿元，公路通车总里程达到1.6万公里。建成高速公路217.2公里，高速公路密度位居全省前列。核电码头、葛洲坝水泥码头、临江山物流园码头等建成运行，通江达海优势日益显现。嘉鱼长江大桥开工建设，多年期盼的大桥梦变为现实。城际铁路率先开通，高速公路县县畅通，城区外环全线贯通，“中三角枢纽地位”初步形成。城市品质大幅提升。十六潭公园、青少年宫、嫦娥广场等对外开放，博物馆、竹博览馆、潜山古寺等相继建成，香城泉都品牌越擦越亮，荣膺国家园林城市、国家卫生城市、国家森林城市等11项金字招牌。县市城区面貌发生巨变，辐射能力不断增强。全市城市建成区面积突破160平方公里，城镇化率提高到51.2%。镇村建设步伐加快。出台全域新型城镇化总体规划，大力推进咸嘉生态文化城镇带和幕阜山绿色产业带建设。获批国家重点镇11个，省级重点镇、特色镇15个。贺胜金融小镇开街，潘家湾镇“四化同步”形成示范。11个村庄被列入全国传统村落保护名录。咸宁被评为全国美丽乡村建设示范区。完成373座病险水库除险加固和27条中小河流治理。“三万”活动扎实开展，农村环境焕然一新。

（三）经济质效大提升。以“竞进提质、效速兼取”为主线，做大总量、提升质量，奋力推进产业发展。工业崛起势头强劲。重点推进“一工程三计划”。六大支柱产业发展壮大，占全市规模以上工业总量73.3%。完成技改投资344.3亿元，年均增长19.7%。战略性新兴产业加快发展，占全市比重提升至13.2%。高新技术企业突破150家，增加值达100亿元。十大产业纳入全省重点成长型产业集群。一大批重大项目如华润电厂二期、奥瑞金二期、金盛兰冶金、瀛通电子、洪盛模具、兴民钢圈、玉龙机械等建成投产。县级以上工业园区企业600家，园区工业总产值占全市总量78.5%。咸宁高新区申报国家级高新区进展顺利，赤壁、通城经济开发区获批省级高新区。现代农业不断壮大。特色农业产业化基地366万亩，高标准农田56万亩，规模以上农产品加工企业294家，农产品加工业产值与农业总产值之比2.5:1。羊楼洞茶业获批国家级农业产业化龙头企业，实现了零的突破。成功创建国家级楠竹生产综合标准化示范区，通城获批国家油茶产业示范园，赤壁获批国家有机产品认证示范区。获得中国驰名商标17件，国家地理标志商标15件，“三品一标”441个，全国“一村一品”示范村9个。第三产业加快发展。实施旅游跨越战略。2016年接待游客4750万人次，旅游总收入达到243亿元，五年年均增长25.9%，跨入全省第一方阵。连续成功举办“国际温泉文化旅游节”，荣获首批全国旅游标准化示范城市、首批国家级旅游改革创新先行区。电子商务发展迅猛，通山、赤壁电子商务进农村综合示范全省领先，绿购网成为全省电子商务示范创建企业。商贸物流加快发展，全社会货运量突破1亿吨。中华鸡汤小镇开业，沃尔玛、大润发等知名企业入驻。

（四）发展动能大增强。以体制机制创新为重点，深化改革创新和对外开放，奋力推进动能转换。改革红利不断释放。深入推进“放管服”改革，市级行政许可服务事项下降到590项，精简率55.2%。咸宁高新区体制机制改革顺利完成，供给侧结构性改革“六大专项行动”扎实推进，“营改增”、商事制度、农村产权制度、小型水利管护机制、医药卫生体制等改革深入开展，政府机构改革、国有企业改制、事业单位分类改革等稳步实施。财政预算体系建设全省领先，崇阳妇幼卫计资源整合模式全国推广。创新能力不断增强。实施科技创新战略。91项科技成果国内领先，年专利申请量和专利授权量增幅位居全省前列。建成创业孵化基地25个、省级众创空间3家，青年企业中心获评“全国青年创业示范园区”。实施金融领先战略。香城产业引导基金全面启动，129家企业登陆武汉股权交易中心。获批全国社会信用体系建设示范市，连续获评全省金融信用市。实施人才保障战略。被确定为“双回双创”人才工程试点市，“百名专家联百企”人才创新项目获评全省一等奖，博士后、院士专家工作站分别达到15家、36家。合作开放不断深入。与岳阳、九江、安庆达成《咸宁共识》。武咸共建光谷南基地，推进旅游一卡通、环保共治、医疗托管、教育合作。累计引进项目2881个，实际到位资金1221亿元。20多家国内外500强企业、上市公司投资咸宁。成功举办2015国际茶业大会。与100多个国家和地区建立贸易往来，缔结国际友好城市7个。

（五）生态质量大提高。以长江大保护、突出环境问题整治为抓手，走生态优先、绿色发展之路，奋力推进绿色崛起。生态优势更加彰显。成功获批国家生态保护与建设示范区、国家可再生能源建筑应用示范市、全国首批水生态文明试点市、国家森林养生试点市，成功创建崇阳桂花国家森林公园，向阳湖、陆水湖、富水、青山、大溪获批国家湿地公园。累计造林189.8万亩，森林覆盖率达到49.5%，生态环境指标位居全省前列。生态治理更加深入。开展长江大保护，“三禁三治”“三非三水”等行动成效显著。全市主要地表水水质优良率100%，跨界断面和县级以上城镇集中式饮用水水源地水质达标率100%，功能区水质达标率93.8%。完成39个土壤国控点位核查，嘉鱼、崇阳、咸安被授予全国国土资源节约集约模范县。成功创建2个国家级生态村、18个省级生态乡镇、164个省级生态村。生态体系更加健全。率先在全国编制绿色崛起规划，出台一系列加强生态文明建设的政策措施。加强自然资源资产审计，实行环境保护“一票否决”。强化“负面清单”管理，严格执行项目引进“五个一律”制度。实施网格化环境监管，构建督查督办联动机制。

（六）民生福祉大改善。以人民群众的满意感、安全感、获得感为标准，竭尽全力办实事，奋力改善民生福祉。民生保障力度加大。民生支出占一般公共预算支出比重达到74.5%，城镇和农村常住居民人均可支配收入分别达到25738元、12955元，是2011年的1.7倍、2倍。五年新增城镇就业23.4万人，城镇登记失业率控制在2.7%以内。形成“3+X”精准扶贫模式，累计脱贫24.7万人。发放救灾资金2.1亿元，救助群众170多万人次。改造农村危房3.7万户，建设各类保障性住房8.2万套。解决53.4万农村居民和14.4万师生饮水安全问题。在全省率先启动城镇居民大病保险，城乡居民社会养老保险参保104.3万人，综合参保率99%。社会事业全面进步。“全面改薄”工程扎实推进，“联校网教”模式成为全国范例，义务教育均衡发展全部通过教育部评估认定。市实验外国语学校建成开学，咸宁职教集团创建国家示范性职业学校进展顺利，咸宁职院技能大赛获全国一等奖。基层医疗卫生服务体系逐步完善，新型农村合作医疗、村级卫生室标准化实现全覆盖。文化软实力不断提升，“香城大舞台”成为全省优秀文化品牌，“一五一十”工程叫响全国。成功举办首届市运动会和咸宁国际温泉马拉松赛。社会治理不断创新。“平安咸宁”深入推进，社会治安防控体系进一步健全，城区电子监控实现全覆盖，特殊人群管理模式成为全国品牌，社区网格化服务荣获中国城市管理进步奖。“六五”普法顺利完成，崇阳荣获全国“六五”普法先进单位。法治政府建设“十六个率先”全省推广。加强信访维稳、食品药品安全监管、质量监督、安全生产、消防等工作，社会大局和谐稳定。政府公开承诺的民生实事基本完成。

双拥创建、国防动员和后备力量建设进一步加强。对口支援新疆、建始县工作深入推进。妇女儿童、青少年、关心下一代、老龄、残疾人等事业全面发展。对台、侨务、外事、科普、保密、档案、方志、统计、气象、人防、能源、通讯、民族宗教、防震减灾、住房公积金、新闻出版、社科、决策咨询等工作取得新的成绩。

五年来，我们不断加强政府自身建设，努力提高行政效能和服务水平。积极开展党的群众路线教育实践活动、“三严三实”专题教育和“两学一做”学习教育，深入开展“主题党日+”活动，深化“治庸问责”，开展电视问政、民评民议活动，推动作风建设制度化、长效化、常态化。自觉接受人大法律监督、工作监督和政协民主监督，共办理人大代表建议329件（含4件议案），办复率100%，满意率97%；办理政协委员提案606件（含1件建议案），办复率100%，满意率98%。认真履行全面从严治党主体责任，落实“一岗双责”。严格执行领导干部经济责任审计，支持纪检监察和司法机关执纪执法。

各位代表，刚刚过去的 2016年，我市经济社会发展呈现出缓中趋稳、稳中向好的良好态势。预计地区生产总值增长8%，一般公共预算收入增长5.9%，规模以上工业增加值增长7%，固定资产投资增长16%，社会消费品零售总额增长10%，城乡居民人均可支配收入分别增长9.5%、8.5%。换届之年，全市人民战胜了“98+”特大洪灾，打响了灾后重建、脱贫攻坚、经济发展、突出环境问题整治四大战役，实现了经济运行、投资、消费、工业、金融、财政“总体平稳”，新兴产业、投资结构、消费热点、金融业态、对外开放“稳中有进”，生活水平、改革发展、创新创业、民生福祉“不断提升”。完成了省定主要经济指标，实现了“十三五”良好开局，为下一步加快发展奠定了坚实基础。

五年的汗水，五年的辉煌。咸宁，每天精彩；人民，更加自信。五年所取得的成绩，是省委、省政府和市委坚强领导的结果，是市人大、市政协监督支持的结果，汇集了全市人民的智慧，饱含了历届班子的心血，凝结了社会各界的关爱。在此，我谨代表市政府，向全市广大干部群众，向各位人大代表、政协委员和各民主党派、工商联、无党派人士，向离退休老同志，向驻咸解放军、武警消防官兵、公安干警，向中直、省直驻咸和在咸投资兴业的企业家，向所有关心支持咸宁发展的社会各界人士和海内外朋友，致以最崇高的敬意和最衷心的感谢！

五年的发展启示我们：必须坚持党的领导，始终保持政府工作的正确方向；必须时刻谋划发展，以第一力度抓第一要务，升腾发展的强大气场；必须坚守绿色定力，不踩生态红线，推进绿色崛起；必须勇于改革创新，坚决克服路径依赖，让创新源泉不断迸发；必须强化务实重行，形成求真务实、真抓实干的良好作风；必须坚持以人为本，把群众所需作为施政所向，不断增强群众的幸福感和获得感。

同时，我们也清醒地认识到，我市正处在爬坡过坎、转型升级的关键时期。经济规模不大，产业层次不高，县域经济不强；区域发展不协调，中心城区辐射带动力不强；城乡公共基础设施不够完善，公共服务和保障水平较低，扶贫脱贫任务艰巨；少数公务人员责任担当意识不够，适应经济发展新常态能力不足，等等。对此，我们将采取有效措施，切实加以解决。

二、今后五年工作的总体安排

未来五年，我市发展机遇前所未有。“一带一路”、长江经济带、长江中游城市群等重大国家战略的深入实施，有利于我们拓展发展空间、提升发展层次、推进区域合作；中部崛起新规划的实施、湖北自贸区加快建设、武咸同城化全面推进，有利于我们共享政策红利、聚积资源要素、发挥自身优势；国家脱贫攻坚、长江大保护和生态屏障建设，有利于我们补齐发展短板、保护绿色资源、加快绿色崛起；历年打下的基础、不断增强的后发优势和良好的政治生态，有利于我们凝聚改革共识、营造发展氛围、激发干事热情。只要我们抢抓机遇，积极作为，乘势而上，顺势而为，完全能够再上一个新台阶，再创咸宁新辉煌！

今后五年经济社会发展的指导思想是：高举中国特色社会主义伟大旗帜，深入学习贯彻习近平总书记系列重要讲话精神，认真贯彻落实省委、省政府和市委一系列决策部署，坚持以新发展理念为指导，坚持稳中求进工作总基调，坚持以推进供给侧结构性改革为主线，以“一区两带三大四重五城”为主要抓手，加快创新驱动，实现绿色崛起，打造香城泉都，建设中国中部“绿心”和国际生态城市，与全省同步全面建成小康社会，奋力开启基本实现现代化新征程。

今后五年经济社会发展的总体目标是：按照市第五次党代会确定的“一个全面建成，五个全面提升”的目标，力争实现“一个领先、两个确保、三个控制、四个高于、五个更加”。“一个领先”：绿色发展质效全省领先。“两个确保”：确保与全省同步脱贫，确保与全省同步全面建成小康社会。“三个控制”：居民消费价格指数、城镇登记失业率、环保和能耗重点指标控制在省定指标以内。“四个高于”：地区生产总值、固定资产投资、一般公共预算收入、城乡居民人均可支配收入增幅高于全省平均水平。“五个更加”：创新能力更加强劲、区域发展更加协调、生态环境更加优美、开放程度更加广阔、人民生活更加幸福。

各位代表，发展是我们党执政兴国的第一要务，也是解决咸宁一切问题的关键。面对发展不够这个最大的实际，面对人民群众对美好生活的新期待，面对全省各地千帆竞发的态势，我们必须增强发展的紧迫感和使命感，以第一力度抓第一要务，自我加压，负重前行，务实担当，用发展的成果造福于民，以发展的实效取信于民。

未来五年，政府要抓好的重点工作是：

（一）突出项目建设。项目建设是经济增长的驾辕之马，是做大发展底盘的关键所在。积极谋划一批项目，争取项目库达到3.5万亿元规模。着眼扩大需求和改善基础设施，实施一批城市互通、流域治理、农田改造、城市管廊、信息化等重大项目。突出抓好产业投资，围绕传统产业推进技改项目，增强企业内生动力；围绕新兴产业加强招商引资，扩大增量，增强活力；围绕绿色产业做强品牌，推动绿色发展。抓好功能性项目，大力推进图书馆、科技馆、档案馆、群艺馆等建设，提升城市品质。推进民生项目建设，重点加快城乡公共设施、脱贫攻坚、环境治理、文化教育等项目建设。创新投融资方式，加快推进PPP项目落地见效。按照“五个一批”推进项目建设，形成梯队式、可滚动、能持续的项目建设发展链条，力争固定资产投资累计达到1万亿元。

（二）突出新型工业化建设。工业是财富之源，工业强则咸宁强，工业兴则咸宁兴。深入实施“一工程三计划”，建设一批“百亿企业、百亿产业、百亿园区”，打造咸宁工业升级版。用“四新”经济改造传统产业，推进传统产业提能增效。积极实施“中国制造2025咸宁方案”，加快发展智能机电、生命健康、节能环保、电子信息、新能源新材料等战略性新兴产业。实施中小企业成长工程，加快推进“个转企”“小进限”和“小进规”，促进市场主体增量提质。全市工业固定资产投资累计突破3000亿元，规模以上工业企业突破1200家，高新技术产业增加值占规上工业增加值比重达到20％。实施千亿农产品加工业计划，加快新业态开发，推进农村一二三产业融合发展。实施服务业倍增计划，加快发展大旅游、大健康、大文化产业，积极推进金融、物流、商贸等发展，服务业占GDP比重达36%以上。

（三）突出信息化建设。信息化是新生产力的代表，是推动产业转型的强大动力。加快发展信息产业，重点培育汽车电子、电子信息、信息服务等新兴领域。实施“互联网+”行动计划，以信息化提升传统产业，让“互联网+”为实体经济插上腾飞的翅膀，培育市级以上两化融合示范企业110家。加快信息化与城市深度融合，运用互联网思维提升城市管理水平。实施大数据战略，全面提升政府运用大数据服务社会能力。推进社会生活信息化，重点加强“云上咸宁”“教育云”“健康云”“一卡通”等公共服务平台建设，打造智慧咸宁。加强信息基础设施建设，推进光纤到户、无线城市建设、三网融合。建立健全网络风险防范机制，重点做好金融、通信、交通等重点领域安全防护工作。

（四）突出新型城镇化建设。城镇化是现代化的必由之路，是咸宁区域发展最有力抓手。修订全域新型城镇化规划，推进“五城同创”，构建以市区为龙头、县城为支撑、城镇为纽带、村庄为基础的发展格局。加快武咸一体化步伐，实施北部空间计划，将其打造成对接武汉窗口区、新兴产业示范区和绿色崛起先行区。围绕咸嘉生态文化城镇带和幕阜山绿色产业带，打造一批国家级、省级名镇，推进北部城镇集聚发展、南部城镇点轴发展。加强分类指导，推动重大项目市县统筹布局，振兴县域经济。控制城市边界，推进节约集约用地，实行城镇建设用地增加规模与吸纳农业转移人口落户数量挂钩政策。推进以人为核心的城镇化，促进城市基础设施、公共服务和优质资源向农村延伸，全面放开城区、建制镇落户限制，推进公共服务均等化。增强城镇人口聚集能力，实现常住人口城镇化率60%。

（五）突出农业现代化建设。“三农”工作是全党工作的重中之重，是社会稳定的压舱石。扎实推进农业供给侧结构性改革，加快农业现代化步伐，使咸宁农业更强、农民更富、农村更美。转变农业发展方式，大力发展特色农业、观光农业、农产品加工业，农业总产值年均增长4.5%，农产品加工业产值达1300亿元。发展适度规模经营，优化经营结构，新发展家庭农场2000家，农业生产综合机械化水平达到78%。加快种养结构调整，以市场需求为导向，提高优质农产品供给能力，新增“三品一标”100个。加快农村体制机制创新，推进农村产权制度改革，完善农村社会化服务体系，不断优化农业政策供给、制度供给。开展美丽宜居乡村建设试点，统筹城乡基础设施布局和建设，加快农村基层公共服务能力提升，建成美丽宜居示范村300个。

（六）突出生态文明建设。绿色是发展之基、咸宁之魂，保护好绿水青山就是保护好生产力。积极创建生态文明建设示范市，确保空气质量位于全省前列、地表水三类以上水质达到100%、森林覆盖率达到53%以上。强化精细化管理和指标刚性约束，推行清洁生产，鼓励企业改进工艺，更新设备。推进产业园区转型升级，建设循环经济示范区、低碳经济示范区。实现村庄保洁体系、乡镇垃圾收集中转设施、县级垃圾无害化处理设施全覆盖，农村生活垃圾治理处理率达到90%以上。实施建设用地总量与开发强度“双控”行动，实现耕地保有量19万公顷以上，新增建设用地规模控制在14.6万亩以内。建设生态文明制度，构建多元参与、激励约束并重、系统完整的生态文明制度，打造生态文明咸宁样板。

（七）突出人民至上。民心是最大的政治，民心必须靠民生来保障。把精准脱贫作为头等大事，实现38.3万贫困人口脱贫、192个贫困村出列。把教育作为民生要事，促进基础教育优质均衡发展，普及高中教育，构建现代职业教育体系，推动高等院校内涵式、应用型发展。把就业作为民生之本，五年完成新增城镇就业25万人，城镇登记失业率控制在4.5%以内。积极推进收入分配制度改革，千方百计增加居民实际收入。提高社会保障统筹层次和水平，城乡基本医疗和养老保险参保率均不低于95%。实施“健康咸宁”战略，推动医养结合，倡导全民健身，全市人均期望寿命提高到78岁。实现农村饮水安全全域性全覆盖。推动房地产长期稳定健康发展。提高公民科学素质。深化平安咸宁建设，完善立体化、数字化社会治安防控体系，严守安全生产和食品药品红线。

三、2017年政府的主要工作

五年宏图，重在开局。2017年，我们将认真贯彻落实中央、省委经济工作会议以及市第五次党代会精神，坚持稳中求进工作总基调，坚持以深化供给侧结构性改革为主线，坚持创新驱动、绿色崛起，以“三抓一优”为重点，全面做好稳增长、促改革、调结构、惠民生、防风险各项工作，推动经济社会提速、进位、增效，以优异成绩迎接省第十一次党代会和党的十九大胜利召开。

今年经济社会发展的主要预期目标是：地区生产总值增长8.5%左右；规模以上工业增加值增长8%；固定资产投资增长14%左右；居民消费价格涨幅控制在3.5%以内；社会消费品零售总额增长11.5%左右；一般公共预算收入增长8.5%；外贸出口总额增长10%左右；城乡常住居民人均可支配收入增长9%左右；城镇登记失业率控制在4.5%以内；完成省定节能减排任务。

重点做好以下七个方面的工作：

（一）扩大投资消费，保持经济平稳健康发展。投资是稳增长的定海神针。强化有效投资、加强招商引资、推进消费升级，助力经济社会行稳致远。

强化投资主引擎。坚持“一季一调度”，形成倒逼机制，确保固定资产投资完成1600亿元。开展“喜迎十九大、实现开门红”系列活动，一季度实施“双百会战”，形成抓投资、抓项目的强大气场。实施“211”重大项目工程，推进20个产业项目、10个基础设施项目、10个文化旅游服务业项目开工建设。全面启动咸宁通用机场、潘家湾港区通用码头等“十三五”重大规划项目，突出抓好南玻光电导光板、立邦涂料全球标杆工厂、武深高速公路嘉鱼北段、咸宁际华国际旅游、咸宁核电等重点项目。积极争取国家和省预算内资金、国家专项建设基金、国家政策性银行贷款资金和省长江经济带产业发展基金等。开展“PPP项目落地年”活动，营造良好投资环境，引导民间投资预期，全力激活民资。

吹响招商集结号。开展“重大项目推介”“招商引资月”等活动，在引进世界500强企业、上市公司或行业龙头上实现新突破，全年完成招商引资1000亿元。突出产业招商、精准招商、基金招商，重点围绕六大绿色产业和“一区两带三大”开展招商。在珠三角、长三角、环渤海等区域组织经贸洽谈会，积极承接发达地区产业转移。坚持全市“一盘棋”，建立定期通报及调度机制，完善招商项目信息库，推动县市联动招商。调优配强招商专班力量，鼓励优秀人才与后备干部投身招商一线。坚持领导带头招商，强化考核与激励，优化招商环境，营造浓厚招商氛围。

释放消费新需求。以新消费引领新供给、培育新动力，加快消费升级步伐。深入推进“十大扩消费行动”，大力发展旅游、文化、健康、养老、体育五大幸福产业，扩大智能终端、智能服务等信息消费，推动线上线下互动消费。发展汽车后市场，引进培育一批汽车综合体，加快建设二手车交易市场。推动电商、网购和快递融合发展，支持咸宁电商产业园、绿购网、世界茶商网建设，支持通城创建国家电子商务进农村综合示范县。推进便民消费进社区、便民服务进家庭“双进工程”，打造15分钟便民生活服务圈。加强市场监管，保持物价稳定，呵护优质品牌，打造诚信咸宁。

（二）实施创新驱动，着力培育发展新动能。创新是引领发展的第一动力，坚持技术、人才、资本齐头并进，推进发展从要素驱动向创新驱动转变。

推进科技创新。抢抓武汉东湖国家自主创新示范区建设机遇，突出咸宁高新区技术创新龙头地位，加快启迪之星孵化基地建设，打造光谷南科技城、津晶城科技园。完善“1+5+X”协同创新体系，推动企业创新主体建设，发挥湖北科技学院、咸宁职院等创新源头优势。大力实施科技创新“五大工程”，力争专利申请量突破2000件，新认定高新技术企业达到30家，高新技术产业增加值增长10%，占GDP比重突破10%。鼓励支持企业建设工程技术研究中心、校企共建研发中心、产业技术研究院、重点实验室等技术研发机构。

强化金融创新。加快贺胜金融小镇建设，全力打造华中私募聚集地、中国特色金融小镇，力争落户基金公司突破100家、管理资金突破300亿元。完善金投集团服务功能，推动金融产品创新，扩大“政银保集合贷”“裕农贷”“创业贷”等金融产品覆盖面，做好“开发担保贷”“公积金安居贷”等业务。扩大直接融资，力争瀛通电子主板上市，挂牌“新三板”企业达到10家，挂牌“四板”企业达到200家。放大香城产业基金规模效应，促进基金、产业、人才融合发展。加强与国内名牌大学合作，加快建设咸宁金融信息港，推进市域金融工程。全力服务实体经济，及时防范金融风险。

聚集创新人才。启动“南鄂英才计划”，更加主动地引进行业领军人物和人才团队，汇聚新经济发展的“众力量”。开展“双回双创”试点，推进“双招双引”“招硕引博”等工程，吸引优秀企业家和大学生返乡创业。开展创业先锋行动，推进专业技术人员知识更新、高技能人才培养、金蓝领开发工程。建成人才创新创业超市，推广“星期六工程师”“候鸟工程师”等柔性引才模式，实现“科研在高校、创业在咸宁”。大张旗鼓表彰创业明星、优秀企业家，努力营造崇尚创新创业的浓厚氛围。

（三）深化改革开放，不断释放发展红利。改革开放是推动发展的关键一招，坚持以改革为引领，以开放为先导，不断增强发展的内生动力和活力。

深化供给侧结构性改革。落实“三去一降一补”任务。关闭6万吨高瓦斯矿井，淘汰煤炭产能12万吨。加大棚户区改造力度，推进租购并举，推动商品房供给结构优化，确保商品住房库存消化周期控制在10个月左右。纵深推进清费减负工作，合理降低企业综合成本，引导企业向内挖潜降成本。积极化解企业融资和政府债务风险，加快发行地方置换债券，推进企业上市融资。申报发行企业债券30亿元。落实城市地下综合管廊、湖泊综合治理、农村小型水利工程等“补短板”行动方案。

推进重点领域改革。清理和规范政府定价的经营服务性收费，确保清单之外无收费。建立行政审批事中、事后分段监管责任制，推进综合行政执法改革，实现“双随机、一公开”监管全覆盖。完善“互联网+政务服务”，发挥“云上咸宁”作用，打破部门数据“孤岛”。深化“五证合一、一照一码”登记制度改革，全面推行“一套材料、一表申请、一窗受理”便民模式。深化农村土地制度改革，推进农村土地“三权分置”。深化财税体制改革，加大县级财政资金统筹力度，提高资金使用效益。深化国有企业改革，推动国有资本合理流动、配置优化。扎实推进供销社综合改革。完成国有林场改革主体工作。全面深化教育、医药卫生、收入分配、社会保障等民生领域改革。

扩大对内对外开放。积极融入武汉城市圈和长江中游城市群，加快武咸同城化步伐，推进咸岳九“小三角”合作，深入推进“通平修”次区域合作，全力打造长江中游城市群合作发展先行区。加快推进嘉鱼长江大桥、赤壁长江大桥、武咸快速公路、106国道和360省道改扩建等项目建设，积极争取武厦高铁咸宁段、岳咸九铁路早日规划建设。深度推进与俄罗斯、蒙古国及中亚各国合作交流，着力构建“一带一路”内陆重要节点城市和万里茶道源头城市。加强与韩国太白市和义王市、匈牙利威莱士艾及哈兹市、法国奥贝维利耶市等国际友城经济文化互动，加快建设中瑞咸宁示范项目、“法国之家”。积极策应湖北自贸区建设，实施外贸转型升级工程，支持名特优产品参加境外国外展销活动。

（四）加快转型升级，进一步优化产业结构。产业是发展的第一支撑，坚持做强传统产业，拥抱“四新”经济，着力构建主导产业“顶天立地”、中小企业“铺天盖地”的发展格局。

做强新型工业。实施工业崛起战略，打造大园区、发展大产业、培育大企业，全面振兴实体经济。推进高新区“满园工程”，全年新投产、开工、签约项目110个，新增规上企业12家、高新技术企业10家。实施传统产业改造升级工程，加快企业研发中心建设，加强全面质量管理，增强产品竞争力，工业企业技改投资增长15%。实施新兴产业培育计划，大力发展新能源、新材料、环卫设备、健康医药等产业，做大做强海威材料、合加环保、海王医药等企业。实施中小企业成长工程，狠抓企业上规入库，积极培育新的经济增长点，全市新增规上企业60家。

发展现代农业。加快农产品千亿工程冲刺步伐，壮大羊楼洞茶业、黄袍山油茶、精华纺织、巨宁竹业等行业龙头，新增省级龙头企业5家，农产品加工业产值突破850亿元。积极创建国家农业可持续发展示范区，推进茶叶、油茶、楠竹、水产等产业发展，打造青砖茶、本草天香、桂花系列产品等品牌。推动农业供给侧改革，发展“订单农业”，培育职业农民、家庭农场、专业大户、农民专业合作社等新型主体，发展休闲农业、设施农业、智慧农业等新业态。全面完成全域永久基本农田划定工作。健全现代农业科技创新推广体系、农业社会化服务体系，提高农业现代化水平。深化“农商对接”“农超对接”，推进“互联网+农业”，拓宽农产品销售渠道。

壮大第三产业。加快发展大旅游、大健康、大文化和商贸物流业，确保服务业占GDP的比重达35%。全面推动旅游业改革创新先行区建设，加快实施“十大旅游发展工程”，着力延伸产业链条，培育核心品牌，力争全市旅游总收入达到280亿元以上。发挥国家森林养生试点市、国家医养结合示范区品牌优势，推进梓山湖大健康产业示范区、华中健康养生谷、药姑山中医药健康科技产业城等项目建设。实施文化项目带动工程，重点打造文化休闲、文化创意、影视娱乐等文化产业，建设一批富有特色和影响力的文化产业基地。优化物流产业布局，建设一批商业中心、特色商业街、乡镇商贸中心等，打造湘鄂赣边贸市场，建设鄂南现代物流园、嘉鱼农产品冷链物流集散中心。引进发展区域性总部经济、服务外包等新兴业态。

（五）推进城乡统筹，进一步优化发展格局。城乡统筹是协调发展的重要内容，坚持城乡互动、五城同创，优化资源配置，构建区域协调发展的新格局。

优化空间布局。启动新一轮城市总体规划修编，优化中心城区和县市区空间格局。出台北部空间规划，引导城市北拓西展。实施107国道外迁、梓山湖大道西延伸、旗鼓大道北延伸等交通工程。以桂乡大道为发展轴，推进高端产业、环保产业、创意产业等聚集发展。加快建设大洲湖湿地公园。推进高新区产城融合，均衡布局学校、医院、金融、商务等公共服务设施，完善城市道路、污水处理、综合管廊等基础设施，推进“一港两城五园”建设。支持县市区按各自定位突出优势、错位发展、争先进位。建设咸嘉生态文化城镇带和幕阜山绿色产业带，推进贺胜桥、横沟桥、潘家湾、大畈、铜钟、麦市等重点镇建设，构成“两带牵引、多点支撑”发展格局。

提升城市品质。实施中心城区品质提升工程。加快城市街心小公园、小游园、小球场建设，改善城区交通微循环，点亮“两山一河”。启动怀德路向西跨淦河至宝塔大道、城北路向北至107国道延伸工程，推动城区向北拓展。实施城市功能提升工程。加快游泳馆、图书馆、档案馆、工人文化宫以及公共停车场、城市绿地等公共设施建设，推进贺胜路、光谷南路地下管廊建设，启动梓山湖海绵城市示范点建设。加快城区防洪工程建设。实施城市文化提升工程。加快建设永安阁、冯京公园等“城市符号”，让建筑刻录城市的年轮。开展国家电信普遍服务补偿机制试点，推动宽带提速降费。实现中心城区主要公共区域免费WiFi全覆盖。推进城管力量下沉，建设数字城管，实现精细、精准、长效管理。

建设美丽乡村。统筹推进秀美香城、鄂南风情小镇和美丽乡村建设，推动城市基础设施、公共服务向基层延伸，实现城乡融合发展。深入实施百村示范、农民安居、环境整治、完善提质四大工程，抓好12个省级美丽乡村示范村建设，打造一批精品样板乡村。强化农村环境综合治理，推行垃圾就地分类减量和资源回收利用，探索农村污水生态化处理。加强农村住宅规划建设工作，综合整治违法占地、违规建房。依托村庄风貌特色、历史文化资源，建设鄂南特色的民居名村。实施“乡土文化百人工程”，发掘、保护和传承咸宁农村特色文化。发展乡村旅游、农耕体验、乡村手工艺等新业态，促进农民增收。

（六）守护绿水青山，厚植绿色发展优势。生态是咸宁最宝贵的财富。必须坚守绿色定力，保护生态环境，发挥绿色优势，在绿水青山中打造金山银山。

加强生态治理。实施“蓝天工程”。严格落实“气十条”，强化“三禁三治”，推动大气联防联控，着力改善大气质量，确保空气质量优良率达到75%以上、PM10浓度均值稳定在70微克/立方米以下，空气质量位居全省前列。实施“碧水工程”。严格落实“水十条”，全面推行“河（湖）长制”，开展“三非三水”治理，全面启动乡镇污水治理工作，全市主要地表水水质优良率100%，跨界断面和县级以上城镇集中式饮用水源地水质达标率100%。实施“净土工程”。严格落实“土十条”，编制《咸宁市土壤污染防治实施方案》，开展土壤污染状况调查，加强土壤环境监管和修复治理，确保耕地土壤环境质量达标率不低于90%。

推进生态建设。修订完善城市生态红线、湖泊保护等专项规划，严格执行城市山体保护规划，加快生态文明示范市创建步伐。推进国家生态保护与建设示范区、水生态文明试点市等试点示范建设，加强市区水体治理力度，创建孝子山国家森林公园，建设10个生态乡镇、100个生态村。积极发展核能、风能、水力发电、生物质发电等清洁能源，推进重点行业能效提升和节能减排，争创国家级环保模范城市。严控城市边界，推进国土整治和资源集约节约利用，提高土地利用率。推广绿色建筑，发展绿色交通，营造绿色消费环境，倡导绿色低碳生活。

筑牢生态屏障。落实国家主体功能区规划，制定实施《咸宁市生态功能区划》，加快构建“两带两河两湖”生态屏障。落实长江大保护职责，重点推进余码头二站、陆水干流防洪治理、斧头湖西凉湖湖堤加固工程建设，确保“满城净水入江，一江清水东流”。实施幕阜山生态保护和修复，全面停止天然林商业采伐，大力推进省级森林城镇、绿色示范乡村创建。支持通城、通山建设国家重点生态功能示范区。积极开展荒山造林和通道绿化，完成造林38万亩，确保“绿满鄂南”行动三年结硬账。

（七）增进人民福祉，不断加强民生建设。人民对美好生活的向往就是我们的奋斗目标，必须坚持以人民为中心的发展思想，让发展成果惠及更多群众。

实施精准脱贫。突出“3+X”产业扶贫，推广“合作社、龙头企业、能人大户、现代服务业+贫困户”四种带动模式，着力增强贫困村和贫困户的自我造血能力。创新搬迁扶贫，推动安居点基础设施建设和脱贫产业培育“双轴并进”，易地搬迁8448户、24291人。实施贫困户子女“9+3”免费教育，阻断贫困代际传递。推进健康扶贫，实施农村医疗精准救治“五大工程”，对所有建档立卡贫困人口参加城乡居民医疗保险实行补贴，确保参保率达到100%，为贫困人口购买商业补充医疗保险。推进项目扶贫，坚持项目向贫困村倾斜、资金向贫困村聚拢，着力改善贫困村生产生活条件。开展金融帮扶，建立完善风险补偿金，充分调动银行信贷积极性，着力解决贫困地区农户和企业贷款难问题。开展“三扶一助两促进”活动，激发群众内生动力，促进精神脱贫。确保10万贫困人口脱贫，103个贫困村脱贫出列。

兜牢民生保障。坚持以创业带动就业，发放创业担保贷款1.8亿元。开展就业专项服务，统筹推进高校毕业生、农民工、零就业家庭等重点群体就业。建立被征地农民基本养老保险补偿机制，全面落实被征地农民社会保障政策。加强社会救助与物价上涨挂钩联动机制，与其他社会保障制度、专项救助与低保救助统筹衔接，构建综合救助工作格局。巩固和完善住房保障体系，加快推进城市棚户区和农村危旧房改造，加快培育和发展住房租赁市场，多渠道解决城乡低收入家庭住房困难。

发展社会事业。实施教育质量提升工程，推动教育全面协调发展。优化教育布局，缓解择校热和大班额问题。市城区新建2所小学和2所幼儿园。推进医疗、医保、医药“三医”联动，突出抓好“两控四改”试点，实现卫生计生服务“一卡通”。发展老年医疗等健康服务业，促进“医养结合”。积极实施全面“两孩”政策，依法打击“两非”行为，综合治理出生人口性别比偏高问题。扎实开展全民健身活动，组织国际温泉马拉松赛、崇阳山地马拉松赛、特殊人群运动会等体育赛事，加强城乡公共健身场所建设。完善公共文化服务体系，实施文化精品工程，支持嘉鱼、崇阳创建省级公共文化服务体系示范区。深入推进公民道德建设，不断提升市民文明素质。

创新社会治理。全面开展“七五”普法，提高社会法治意识。建立多途径、多层次矛盾化解机制，引导群众合理合法表达诉求。深入实施“天网、雪亮、地网、群防”四大工程，提高“一感两度两率”，建设更高水平的平安咸宁。强化社会信用体系建设，建立信用信息共享机制和失信惩戒制度。加强重点行业、重点领域安全隐患排查整治，加强职业病防治，坚守安全生产红线。加强食品药品监督管理，创建国家食品安全示范城市，切实保障人民群众“舌尖上的安全”。积极开展双拥模范城创建活动，统筹推进国防动员、民兵预备役、武警、应急动员等工作，推进军民深度融合。

今年继续办好政府十件实事：（1）全市城镇新增就业3.5万人，城镇登记失业率控制在4.5%以内。（2）新开工棚户区改造11219套，基本建成1845套。（3）全市建设食品追溯体系单位或名厨亮灶示范单位600家，市城区建成1个日处理能力约100吨的餐厨垃圾无害化处理场所。（4）实施交通安全生命防护工程，整治交通安全隐患800处，改造农村危险路段1000公里。（5）建立机关事业单位与企业职工相同的统帐结合的基本养老保险制度，整合城乡居民基本医疗保险制度。（6）提高全市80岁以上老人高龄补贴标准，80-99岁60元/人月；100岁以上300元/人月。（7）实施从南川水库、鸣水泉到双鹤桥32公里淦河综合整治工程，完成三分之一建档立卡贫困户饮水安全问题。（8）新建村级邮政站所200家。（9）实现农村宽带全覆盖、光纤村村通。（10）完成西凉湖、斧头湖17.4万亩围网围栏拆除。

四、加强政府自身建设

今年是政府换届后的起始之年，发展的任务很重，必须全面加强政府自身建设，不断提升履职尽责能力。

建设忠诚政府。牢固树立“四个意识”，始终在思想上政治上行动上与以习近平同志为核心的党中央保持高度一致，确保对党绝对忠诚。坚决把贯彻落实党中央国务院和省委省政府重大决策部署作为首要政治任务，不打折扣、不搞变通，确保政令畅通、执行有力。坚决在市委领导下开展工作，提高保落实的能力，确保市委的意图和决策全面贯彻到政府工作中。

建设责任政府。始终把推动发展作为主职主责，不搞形式主义、不搞弄虚作假。把重大机遇、重大决策、重要工作落实到项目上、落实到工程上。建立容错纠错机制，旗帜鲜明地为敢于担当者担当、为敢于负责者负责，让干部想干事、敢干事、干成事。建立正向激励机制，鼓励干部在一线干出实绩、展现作为。不断提升干部科学思维的能力、依法行政的能力、服务群众的能力，以政府的高效率换取发展的加速度。

建设法治政府。树立法治信仰，落实宪法宣誓制度。严格落实公众参与、专家论证、风险评估、合法性审查、集体讨论决定等法定程序。加快建立统一的行政执法监督网络平台，建立健全投诉举报、情况通报等制度。自觉接受人大、政协和人民群众监督，促进权力规范运行。推进全民普法守法，形成办事依法、遇事找法、解决问题用法、化解矛盾靠法的良好环境。

建设廉洁政府。落实全面从严治党主体责任清单，加强政府自身建设。强化行政审批和土地交易、建设工程、政府采购、产权交易等重点领域和关键环节的监管。推进重大决策、重点工作、重要安排及时公开，阳光透明。树立“过紧日子”思想，提高财政资金使用绩效，确保“三公”经费“零增长”。持续开展反“四风”，积极整治乱作为、不作为、慢作为，努力打造清正、廉洁、阳光政府形象。

各位代表，一代人有一代人的担当，一代人有一代人的使命。只有与历史同步伐、与时代共命运，才能赢得光明的未来。让我们紧密团结在以习近平同志为核心的党中央周围，在省委、省政府和市委的坚强领导下，在市人大及其常委会的有力监督下，在市政协的大力支持下，不忘初心，继续前进，为加快实施创新驱动、绿色崛起战略，与全省同步全面建成小康社会，全面开启基本实现现代化新征程而努力奋斗！
